# COVID-19: Healthcare Workers May Be at Greater Risk Outside Their Work Environment—A Retrospective Observational Study

**DOI:** 10.5041/RMMJ.10469

**Published:** 2022-04-26

**Authors:** Khetam Hussein, Yael Shachor-Meyouhas, Halima Dabaja-Younis, Moran Szwarcwort-Cohen, Jalal Tarabeia, Avi Weissman, Michal Mekel, Gila Hyams, Michael Halberthal

**Affiliations:** 1 The Infection Control Unit, Rambam Health Care Campus, Haifa, Israel; 2 The Ruth & Bruce Rappaport Faculty of Medicine, Technion–Israel Institute of Technology, Haifa, Israel; 3 Pediatric Infectious Diseases Unit, Ruth Rappaport Children’s Hospital, Rambam Health Care Campus, Haifa, Israel; 4 Management, Rambam Health Care Campus, Haifa, Israel; 5 Virology Laboratory, Rambam Health Care Campus, Haifa, Israel; 6 Nursing Administration and Management, Rambam Health Care Campus, Haifa, Israel

**Keywords:** COVID-19, epidemiology, health care workers, infectious diseases, public health

## Abstract

**Background:**

With the availability of coronavirus disease 2019 (COVID-19) vaccine, concerns have been raised regarding pre-vaccination seroprevalence in healthcare workers (HCW). This study examines the seroprevalence of HCW at an Israeli tertiary medical center before first BNT162b2 vaccination.

**Methods:**

This was a retrospective observational study. Before vaccination, HCW at our center were offered serological testing. Data on their epidemiological, workplace, and quarantine history were collected. The SARS-CoV-2 IgG assay was performed pre-vaccination.

**Results:**

A total of 4,519 (82.5%) of the HCW were tested. Of these, 210 were seropositive; 101 had no known history of COVID-19. Of the 101 asymptomatic HCW, only 3 (3%) had worked at COVID-19 departments, and 70 (69.3%) had not been previously quarantined. Positive serology was similarly distributed across age groups, and about 40% had no children. Nearly half of the HCW tested were administrative and service staff. Overall, seropositive tests were associated with having no children (OR 1.42, 95% CI 1.06–1.89; *P*=0.0218), history of having been quarantined without proof of disease (OR 6.04, 95% CI 4.55–8.01; *P*<0.001), and Arab ethnicity (OR 3.36, 95% CI 2.54–4.43; *P*<0.001). Seropositivity was also more prevalent in members of the administration compared to other sectors, medical and paramedical, who are exposed to patients in their daily work (OR 1.365, 95% CI 1.02–1.82; *P*=0.04).

**Conclusions:**

The low percentage of asymptomatic COVID-19 among our HCW may reflect the high compliance to personal protective equipment use despite treating hundreds of COVID-19 patients. The relatively high number of childless seropositive HCW could reflect misconceptions regarding children as a main source of infection, leading to carelessness regarding the need for appropriate out-of-hospital protection.

## INTRODUCTION

Coronavirus disease 2019 (COVID-19) has claimed the lives of more than 5 million people globally, including more than 8,000 in Israel as of November 2021.^1^ Many healthcare workers (HCW) worldwide contracted the disease, and many have died.^2^

There is a high risk for HCW providing direct care to COVID-19 patients, especially if proper personal protective equipment (PPE) is not available or used.^3^–^7^

In mid-December 2020, with the approval of the first vaccines, both management and staff wanted to know the extent of occult severe acute respiratory syndrome coronavirus 2 (SARS-CoV-2) cross-infection. This was particularly the case among HCW who had been working within the healthcare system and already had contact with suspected and proven COVID-19 patients. Furthermore, the actual prevalence of such exposure is most likely lower than perceived by HCW. In a German study, of the 217 HCW surveyed (21%) who expected themselves to be seropositive, seroprevalence was found in only 1%–2%.^8^ Worldwide, SARS-CoV-2 seropreva-lence among HCW varies widely depending on the country and the work setting.^9^,^10^ One study of seroprevalence of frontline HCW found that 8%–31.2% had antibodies, of whom 29% were asymptomatic.^10^ Seropositivity differences were also noted between medical professions and treatment setups.^11^,^12^ Some studies have noted risk factors outside the healthcare system. For example, in India (pre-Delta variant), many of the positive cases were among administrative staff and individuals using public transportation.^13^ In other studies, not wearing a mask and household contact were significantly associated with seropositivity.^5^–^7^

Prior to the first BNT162b2 vaccine dose (Pfizer–BioNTech COVID-19 vaccine, Pfizer Inc., New York, NY, USA), all HCW at our medical center were offered a free serology test to address concerns raised regarding whether or not HCW were already protected after asymptomatic SARS-CoV-2 infection.

This study describes the seroprevalence of blood tests obtained from 85% of our hospital’s staff before vaccination and discusses the impact of our findings.

## METHODS

This retrospective observational study was conducted at Rambam Health Care Campus (RHCC), Northern Israel’s only tertiary academic healthcare facility serving a potential population of 2.3 million residents in the region. The hospital employs 5,478 workers from both cities and villages, residing from the center of Israel to the far north. The COVID-19 department at RHCC opened in early March 2020; since then, more than 2,700 patients have been treated in dedicated coronavirus wards, the emergency room, and pediatric, obstetric, and gynecological wards.

At the end of November 2020 all HCW at the hospital were offered a SARS-CoV-2 serology blood test to determine their immunological status before vaccination. Additional data collected from the hospital databases included: age, sex, ethnicity, marital status, parental status, hospital department, work sector (medical, nursing, paramedical/allied health professions, administrative, etc.), known exposure to COVID-19 and/or quarantine, work in COVID-19 departments, and previous confirmed COVID-19 infection.

The study was approved by RHCC ethical committee (ID# IRB 021-021).

### Laboratory Tests

Testing was performed using the SARS-CoV-2 IgG assay (Abbott Laboratories, Abbott Park, IL, USA). Confirmation of positive serology in personnel was performed using COVID-19 S1/S2 IgG (DiaSorin, Saluggia, Italy). An IgG level above 1.4 AU/mL was considered positive. All HCW had a negative PCR at the time of the blood tests.

Patient characteristics were summarized using descriptive statistics. We calculated the proportion of seropositive patients out of the total number of HCW recruited for this study. The proportion of asymptomatic seropositive subjects was calculated out of the total number of HCW who had not been previously diagnosed with the disease. Differences in frequencies of the different demographic variables between seropositive and seronegative HCW were evaluated with the aid of the chi-square test using a dedicated statistical software (IBM SPSS Statistics for Windows, version 26.0, released 2019; IBM Corp., Armonk, NY, USA). Odds ratios and 95% confidence intervals (95% CI) were calculated. All statistical tests were two-tailed; *P*≤0.05 was considered statistically significant.

## RESULTS

A total of 4,519 of 5,478 RHCC HCW (82.5%) were tested for SARS-CoV-2 antibodies using the above-mentioned immunoassays; 210 HCW were seropositive, of whom 109 had been previously diagnosed with COVID-19. The seroprevalence among HCW who had not been previously diagnosed was 2.3% (101/4410). Of the unknown seropositive cases, only 3 (3%) had worked at the COVID-19 patient wards, and 31 (30.4%) had been quarantined before the positive serology test.

Table 1 shows the characteristics of seropositive HCW with no documented previous infection. In particular, note that 59.6% of the HCW had children and 41.6% had children under the age of 18 years. More than 45% were administrative workers, while nurses and physicians accounted for 22.8% and 13.9%, respectively.

**Table 1:** Characteristics of Seropositive Healthcare Workers with No Known Past Confirmed Infection.

Characteristic	Number	Percent
Sex		
Male	44	43.6
Female	57	56.4

Age Group		
≤30	17	16.8
31–40	31	30.7
41–50	24	23.8
51–60	19	18.8
>60	10	9.9

Marital Status*		
Married	55	54.5
Widowed/divorced	7	6.9
Single	38	37.6

Parenting Status		
No children	41	40.6
With children	60	59.4

Children <18 Years		
No	59	58.4
Yes	42	41.6

Ethnicity		
Jewish	49	48.5
Arab	52	51.5

Past Quarantine		
No	70	69.3
Yes	31	30.7

Working in COVID-19 Wards		
No	98	97
Yes	3	3

Sector†		
Administration and service	46	45.5
Nursing	23	22.8
Paramedical/allied health professions	16	15.8
Physicians	14	13.9

* Data missing for 1 healthcare worker.

† Data missing for 2 healthcare workers.

The characteristics in seropositive and seronegative HCW are presented in Table 2. Seropositive tests were associated with having no children (OR 1.42, 95% CI 1.06–1.89; *P*=0.022), history of having been quarantined without proof of disease (OR 6.04, 95% CI 4.55–8.01; *P*<0.001), and Arab ethnicity (OR 3.36, 95% CI 2.54–4.44; *P*<0.001). We already described that administration represented the biggest sector among those with seropositive HCW with no documented previous infection (Table 1). Comparison of administration to the other sectors, all of whom are exposed to patients in their daily work, reveals that seropositivity was more prevalent in this sector (OR 1.37, 95% CI 1.02–1.82; *P*=0.04). Other characteristics were not associated with seropositivity. Of note, differences observed in seropositivity rates between those who worked in COVID-19 wards and those who did not were not significant (*P*=0.134).

**Table 2:** Characteristics of Seropositive and Seronegative Healthcare Workers.

Characteristic	Positive Serology *n* (%)	Negative Serology *n* (%)	*P* Value	OR (95% CI)
Sex				
Male	91 (5.4)	1589 (94.6)	0.059	
Female	109 (3.8)	2730 (96.2)		

Age Group				
≤30	30 (5)	567 (95)	0.136	
31–40	69 (5.6)	1172 (94.4)		
41–50	54 (4.8)	1074 (95.2)		
51–60	39 (4.1)	909 (95.9)		
>60	18 (3)	587 (97)		

Marital Status*				
Married	125 (4.3)	2782 (95.7)	0.125	
Widowed/divorced	20 (4.2)	456 (95.8)		
Single	64 (5.8)	1045 (94.2)		

Parenting Status				
No children	77 (5.8)	1251 (94.2)	0.018	0.70 (0.53–0.94)
With children	133 (4.2)	3058 (95.8)		

Children <18 Years				
No	117 (4.8)	2307 (95.2)	0.537	
Yes	93 (4.4)	2002 (95.6)		

Ethnicity				
Jewish	100 (3)	3246 (97)	<0.001	3.36 (2.54–4.44)
Arab	110 (9.4)	1063 (90.6)		

Sector				
Administrative/service	77 (5.7)	1283 (94.3)	0.040	1.37 (1.02–1.82)
Healthcare providers†	133 (4.2)	3026 (95.8)		

Past Quarantine				
No	99 (2.7)	3634 (97.3)	<0.001	6.02 (4.55–8.00)
Yes	111 (14.1)	675 (85.9)		

Working in COVID-19 Wards				
No	199 (4.6)	4166 (95.4)	0.134	
Yes	11 (7.1)	143 (92.9)		

* Data missing for marital status: positive serology, *n*=1; negative serology, *n*=26.

† Includes physicians, nurses, and paramedical staff.

CI, confidence interval; *n*, number; OR, odds ratio.

## DISCUSSION

This study was conducted in an Israeli tertiary medical center and described the pre-COVID-19 vaccination status of HCW who had not previously been identified as COVID-19 positive. The low sero-prevalence (101/4519, 2.23%), might indicate the importance and success of appropriate PPE use and adherence to infection control measures in the management of COVID-19 suspected or confirmed patients. Most of the asymptomatic HCW did not recall contact with COVID-19 patients or relatives, which is supported by other studies with few known sources for SARS-CoV-2.^8^,^10^,^14^ Only 11 (5%) of our seropositive HCW had a history of having worked at the COVID-19 departments.

The high percentage of seropositive administrative HCW among those tested may be linked to the high risk of exposure in the community and house-hold, and possibly while using public transportation or hospital transportation to get to work. Similar observations were published by Gupta et al., where HCW using public transportation were more likely to be seropositive.^13^ Another study showed that seroprevalence was higher in ancillary HCW and staff in non-COVID hospitals, stressing the need to wear masks and appropriate PPE use.^11^

This is an important aspect that can be used to boost HCW confidence in the effectiveness of proper PPE use while treating patients with other infectious diseases and offers an opportunity for future studies into effective PPE training methodologies.

We also found that 97% of the tested HCW had not worked in a dedicated COVID-19 ward. It should be noted that the dedicated COVID-19 wards at RHCC have very high standards of implementation and enforcement of infection control guidelines, including education, training, and proper PPE use and donning/doffing while under supervision. The importance of PPE has also been noted in some systematic reviews and a meta-analysis.^5^–^7^ Our hospital also has an infection prevention and control team that educated staff and monitored the use of PPE on a daily basis. In addition, HCW were allowed to work only four cumulative hours per shift in full PPE, and a digital reporting system was used at the entrances and exits for all dedicated COVID-19 wards.

Another interesting observation from our study was the high percentage of seropositive participants without children, despite there being more HCW with children at home and in quarantine. Not having children at home, may have been perceived as a reduced risk for infection, leading to carelessness in social situations on the part of these HCW, even when published infection rates remained quite high. The perception that children are a main source of asymptomatic COVID-19 should be explored and investigated separately.

## LIMITATIONS

Our study had some limitations. First, the generalizability of our findings is unknown, as our study was limited to a single center. Second, this was a retrospective study with the limitations accompanying such a study. Finally, there was no breakdown of the family status (with or without children) of quarantined HCW.

## CONCLUSION

The low percentage of asymptomatic COVID-19 HCW at RHCC may reflect the high PPE compliance from the beginning of the pandemic, as indicated by the low prevalence among HCW who directly treated such patients, despite receiving and treating hundreds of COVID-19 patients. The higher number of quarantined HCW without children may also reflect the out-of-hospital risk, when the use of appropriate protection may not be perceived as necessary. This could point to a need for medical education to target healthcare workers for improved safety compliance and behavioral adaptation in crisis situations.

This study, along with the data from many other studies,^3^–^11^ should be used to educate HCW regarding the need for proper PPE use, to boost their confidence regarding actual personal safety when caring for patients with infectious diseases, and to stress the importance of taking proper safety precautions even when not on duty.

## Abbreviations

COVID-19: coronavirus disease 2019
HCW: healthcare workers
PPE: personal protective equipment
RHCC: Rambam Health Care Campus
SARS-CoV-2: severe acute respiratory syndrome coronavirus 2.
